# Long-term survival and portal vein patency with novel PVTT surgery approach in advanced HCC patients with Vp3/4 PVTT following combination therapy of TKIs and PD-1 inhibitors

**DOI:** 10.1186/s12893-023-02291-1

**Published:** 2023-12-19

**Authors:** Tianyu Jiao, Haowen Tang, Wenwen Zhang, Bingyang Hu, Tao Wan, Yinbiao Cao, Ze Zhang, Yafei Wang, Junning Cao, Mengqiu Cui, Shichun Lu

**Affiliations:** 1 Medical School of Chinese PLA, Beijing, China; 2 Faculty of Hepato-Pancreato-Biliary Surgery, the First Medical Centre, Chinese PLA General Hospital, Beijing, China 100853; 3 Institute of Hepatobiliary Surgery of Chinese PLA, Beijing, China; 4 Key Laboratory of Digital Hepetobiliary Surgery, PLA, Beijing, China; 5 Nankai University School of Medicine, Tianjin, China; 6 Organ Transplantation Center, The Affiliated Hospital of Qingdao University, No. 59 Haier Road, Qingdao, Shandong China; 7 Department of Radiology, the, First Medical Centre , Chinese PLA General Hospital, Beijing, China; 8 Faculty of Hepato-Pancreato-Biliary Surgery, Chinese PLA General, Beijing, China

**Keywords:** Hepatocellular carcinoma, Portal vein tumor thrombus, Salvage surgery, Programmed cell death protein 1 inhibitors, Tyrosine kinase inhibitors

## Abstract

**Background:**

It is controversial whether patients with hepatocellular carcinoma (HCC) with portal vein tumor thrombus (PVTT) should undergo salvage surgery following the combination therapy of tyrosine kinase inhibitors (TKIs) and programmed cell death protein 1 (PD-1) inhibitors. This study aimed to elucidate the efficiency and safety of salvage surgery following combination therapy, while also summarizing a novel surgical approach for Vp3/4 PVTT.

**Methods:**

Between April 2019 and December 2022, a consecutive series of unresectable HCC patients with PVTT who received salvage surgery following combination therapy were enrolled. Evaluation included perioperative and long-term follow-up outcomes. The complete removal of Vp3/4 PVTT was achieved using a novel surgical approach characterized by “longitudinal incision and transverse suturing” and “angle-to-straight conversion”.

**Results:**

Forty patients including 22 patients with Vp3 and 18 patients with Vp4 were included. Long-term follow-up showed similar rates of portal vein patency (Vp3: 95.5%, Vp4:94.4%, *p* = 0.900), and 3-year portal vein patency rates were 95.0%. There were no significant differences observed in combination therapy-related adverse events (*p* = 0.253) and perioperative complications (*p* = 0.613) between the Vp3 and Vp4 groups. The recurrence patterns were similar between the two groups (*p* = 0.131). There were no significant differences in overall survival (OS) and recurrence-free (RFS) survival between the Vp3 and Vp4 groups (OS *p* = 0.457, RFS *p* = 0.985). Patients who achieved a pathological complete response had significantly better RFS (*p* = 0.011).

**Conclusion:**

Salvage surgery after combination therapy demonstrated favorable efficacy and safety. The novel surgical approach for PVTT can effectively achieve complete removal of PVTT and ensured long-term portal vein patency.

## Background

Hepatocellular carcinoma (HCC) is one of the most common malignancies and a leading cause of cancer-related mortality worldwide [1]. In China, the incidence of HCC accompanied by portal vein tumor thrombus (PVTT) was reported to be 44%-62.2% [2]. According to the Barcelona Clinic Liver Cancer (BCLC) classification [3], the presence of PVTT indicates an advanced stage of HCC and is associated with therapeutic challenges and a poor prognosis [4–6], with a median overall survival (mOS) of only 6.1–11.2 months following traditional treatments [7]. These patients were regarded as unresectable in the majority of international guidelines, and systemic treatments such as programmed cell death protein 1 (PD-1) inhibitors and tyrosine kinase inhibitors (TKIs) were recommended [8–10].

Recently, the combination of atezolizumab and bevacizumab has been approved as a novel first-line treatment for unresectable HCC and has improved patient outcomes, with a mOS of 19.2 months, median progression-free survival (mPFS) of 6.8 months, and an objective response rate (ORR) of 35.4% [11, 12]. Another combination of lenvatinib and pembrolizumab for unresectable HCC also showed promising results, with a mOS of 22 months, mPFS of 9.3 months, and ORR of 46% [13]. Consequently, various treatment strategies combining TKIs and PD-1 inhibitors have been evaluated and have shown encouraging preliminary data [14]. Given the high ORR after combination therapy, a proportion of initially unresectable HCC can be converted to resectable HCC, allowing for salvage surgery [15]. Till now, certain studies with limited cases have been reported sporadically [15–21], which suggests that salvage surgery after the combination therapy may be feasible.

However, limited by the sample size of previous studies, little is known about the efficiency and safety of salvage surgery, as well as the surgical approach of PVTT. As early as 2016, the current authors initiated combination therapy based on TKIs and PD-1 inhibitors for unresectable HCC [22]. With accumulating experiences, a consensus on salvage surgery following combination therapy of PD-1 inhibitors and TKIs for advanced HCC among Chinese experts has also been reached and drafted by our team [23]. Based on our long-term clinical practice, the present study aims to report the outcomes of a cohort of patients with initially unresectable HCC with PVTT who underwent combination therapy of TKIs plus PD-1 inhibitors and salvage surgery, as well as to summarize the surgical experience, particularly in the management of PVTT.

## Materials and methods

### Patients

Data from a consecutive series of patients with unresectable HCC with PVTT who underwent salvage surgery after combination therapy of TKIs plus PD-1 inhibitors were analyzed. All patients met the following inclusion criteria: (1) HCC was diagnosed histologically and according to the American Association for the Study of Liver Diseases (AASLD) guidelines [8]; (2) Underwent a salvage surgery after successful combination therapy; (3) Child–Pugh score < 7; (4) BCLC stage C, with PVTT; (5) Eastern Cooperative Oncology Group performance status (ECOG PS) score ≤ 1; (6) expected survival ≥ 12 weeks; (7) absence of esophageal or gastric varicose bleeding events due to portal hypertension in the past 6 months; (8) at least one measurable tumor lesion by the modified Response Evaluation Criteria in Solid Tumors (mRECIST) [24]; and (9) no history of administration of any PD-1 inhibitors, TKIs, or any other immunotherapy. The Exclusion Criteria were: (1) Patients who did not respond to combination therapy; (2) Patients unwilling to undergo salvage surgery; (3) Patients without incomplete clinical, imaging, or survival data.

All patients were divided into Vp3 and Vp4 groups according to the PVTT classification. The present study was performed in accordance with the Declaration of Helsinki and was approved by the Chinese PLA General Hospital Ethics Committee (Approval No. S2018-111–01). All patients signed the written informed consent before the initiation of treatment and salvage surgery.

### Combination treatment

TKIs included lenvatinib (12 mg for body weight ≥ 60 kg, and 8 mg for body weight < 60 kg, orally once a day), sorafenib (0.2 g, orally, twice a day), and apatinib (0.85 g, orally, once a day). PD-1 inhibitors were intravenously administered as follows: sintilimab (200 mg), or nivolumab (3 mg/kg), or camrelizumab (200 mg), or toripalimab (240 mg), or tislelizumab (200 mg), or pembrolizumab (200 mg), every 3 weeks as a cycle. Dose reduction or discontinuation was recommended for patients who experienced serious adverse events (AEs) according to the ASCO guideline [25].

### Surgical information

The outcomes of blood tests and dynamic contrast-enhanced magnetic resonance imaging (DCE-MRI) were assessed preoperatively. Patients who were eligible for salvage surgery met the following criteria: (1) radical resection could be achieved with sufficient remnant liver volume; (2) intact or reconstructable vascular structure of the reserved liver; (3) Child–Pugh score < 7; (4) ECOG PS score ≤ 1; (5) absence of severe AEs due to the combination therapy; and (6) evaluation of main tumor as completed response (CR), partial response (PR) or stable disease (SD) according to the mRECIST for at least 2 months.

In the event of postoperative recurrence, treatment options such as curative resection, radiofrequency ablation, after-line drug therapy, TACE, or best supportive treatment can be considered based on the individual patient's condition.

### Pathological and radiological assessment

All tumor samples were examined by experienced hepatopathologists. In the present study, pathological complete response (pCR) was defined as no residual viable tumor cells from completely sampled tumors. The radiological assessment was performed based on patients’ DCE-MRI at the baseline and every 6–8 weeks after treatment initiation. PVTT was classified according to the Vp grading system [26]. PVTT downstaging is defined as the radiographic observation of regression of PVTT. Tumor response, including CR, PR, SD was assessed according to the mRECIST [24] and evaluated by professional radiologists who were blinded to pathological results.

### Follow-up

The primary endpoint was RFS, which was defined as the time from the salvage surgery to the first radiologically confirmed recurrence or death from any cause. The secondary endpoint was OS, which referred to the time from the start of treatment to death from any cause. All the included patients were treated and followed up regularly. All Patent’s data were systematic collected. The combination therapy related and perioperative AEs were graded using the CTCAE (version 5.0) and the Clavien-Dindo Classification of Surgical Complications [27].

### Statistical analysis

Statistical analysis was performed using SPSS 20.0 software (IBM, Armonk, NY, USA) and R 4.2.1 software. Continuous variables were expressed as median (range) and compared using the student’s t test or the Mann–Whitney test. Categorical variables were presented as frequency (percentage) and compared using the Chi-square test or Fisher’s exact test. Kaplan–Meier curves were generated for OS, RFS, and the log-rank test was used to compare between groups. Differences were considered statistically significant if the p value was lower than 0.05. HRs and their 95% confidence intervals (CIs) were estimated from the Cox model. The statistical power is calculated using GPower 3.1 with α set at 0.05 and β set at 0.2.

## Results

### Patients’ baseline characteristics

A consecutive series of 40 patients (22 patients in Vp3 group, 18 patients in Vp4 group) with initially unresectable HCC and PVTT were enrolled from April 2019 to December 2022. All patients underwent salvage surgery following combination therapy of TKIs and PD-1 inhibitors.

Patients’ demographic and baseline clinical characteristics are summarized in Table 1. The majority of patients with primary HCC had a background of HBV-related cirrhosis, accounting for 90.9% in Vp3 group and 83.3% in Vp4 group (*p* = 0.822). There were no significant differences between the Vp3 and Vp4 groups in terms of demographic characteristics, baseline liver function, alpha-fetoprotein (AFP) levels, tumor number, size, or lymph node metastasis.

**Table 1:** Baseline and perioperative characteristics

Characteristics	Vp3 (*N* = 22) n (%)	Vp4 (*N* = 18) n (%)	*p* value
**Demographic**			
Age, median (range), year	55.0 (38–67)	57.0 (31–68)	0.682
Gender (male/female)	19 (86.4)/ 3 (13.6)	14 (77.8)/4 (22.2)	0.680
Etiology, HBV, yes	20 (90.9)	15 (83.3)	0.822
Anti-viral treatment, yes	13 (59.1)	10 (55.6)	0.822
Liver cirrhosis, yes	20 (90.9)	15 (83.3)	0.642
**Baseline data**			
Child–Pugh score (5/6)	13 (59.1)/ 9 (40.9)	14 (77.8)/ 4 (22.2)	0.209
ECOG performance status = 0, yes	22 (100.0)	17 (94.4)	0.450
Hemoglobin, median (range), g/L	137.5 (114–171)	145 (105–189)	0.189
WBC count × 10^9^/L, median (range)	5.62 (2.29–10.45)	5.75 (3.67–11.99)	0.276
Platelet count × 10^9^/L, median (range)	142 (67–327)	161 (79–312)	0.779
AFP at baseline > 400 ng/ml, yes	10 (45.5)	11 (61.1)	0.726
Tumor number (single/ multiple)	14 (63.6)/ 8 (36.4)	9 (50.0)/ 9 (50.0)	0.385
Tumor diameter > 10 cm, yes	12 (54.5)	12 (66.7)	0.436
Lymphatic metastasis, yes	4 (18.2)	7 (38.9)	0.173
**Perioperative data**			
AFP before surgery > 400 ng/ml, yes	2 (9.1)	6 (33.3)	0.110
Laparotomy operation	19 (86.4)	18 (100)	0.238
Treatment cycle, median (range), time	4.5 (3–23)	5 (3–9)	0.946
Types of hepatectomy (left/right/segmental)	5 (22.7)/6 (27.3)/11 (50.0)	4 (22.2)/8 (44.4)/6 (33.3)	0.465
Hospital stay after surgery, median (range), day	8.5 (5–33)	9.5 (5–20)	0.697
Surgical time, median (range), min	265.5 (178–390)	260 (180–405)	0.697
Blood loss, median (range), mL	375 (50–1000)	575 (50–3000)	0.058
Child–Pugh 5th days after surgery (5/6)	15 (68.2)/7 (31.8)	14 (77.8)/4 (22.2)	0.377

*PVTT* portal vein tumor thrombosis, *Vp* portal vein invasion, *HBV* hepatitis B virus, *ECOG* eastern cooperative oncology group, *WBC* white blood cell, *AFP* alpha-fetoprotein

### Salvage surgery after the combination therapy

The results of preoperative evaluation and surgical features are shown in Table 1. All patients met the above-mentioned criteria. The TKIs were withdrawn 7 days before surgery to minimize their influence on the surgery, while PD-1 inhibitors were continually used during the perioperative period. Preoperative AFP levels exceeding 400 ng/mL were observed in 2 patients (9.1%) in the Vp3 group and 6 patients (33.3%) in the Vp4 group, and the difference was not statistically significant (*p* = 0.110). Laparotomy operation was performed in 19 patients (86.4%) in the Vp3 group and 18 patients (100%) in the Vp4 group, with no significant difference noted (*p* = 0.238). The median number of preoperative drug therapy cycles was 4.5 in the Vp3 group and 5 in the Vp4 group, showing no significant distinction (*p* = 0.946). Types of hepatectomy included 5 left hepatectomies, 6 right hepatectomies, and 11 segmental liver resections in the Vp3 group, while the Vp4 group consisted of 4 left hepatectomies, 8 right hepatectomies, and 6 segmental liver resections, without significant difference between the groups (*p* = 0.465). The median operation time was 265.5 min in the Vp3 group and 260 min in the Vp4 group, with no statistically significant distinction (*p* = 0.697). The median intraoperative blood loss was 375 mL in the Vp3 group and 575 mL in the Vp4 group, showing a marginal difference (*p* = 0.058). Postoperatively, all patients in both groups achieved Child–Pugh grade A scores on the 5th day, without a significant difference observed (*p* = 0.377). Both groups had no mortality during the perioperative period.

**Fig. 1:**
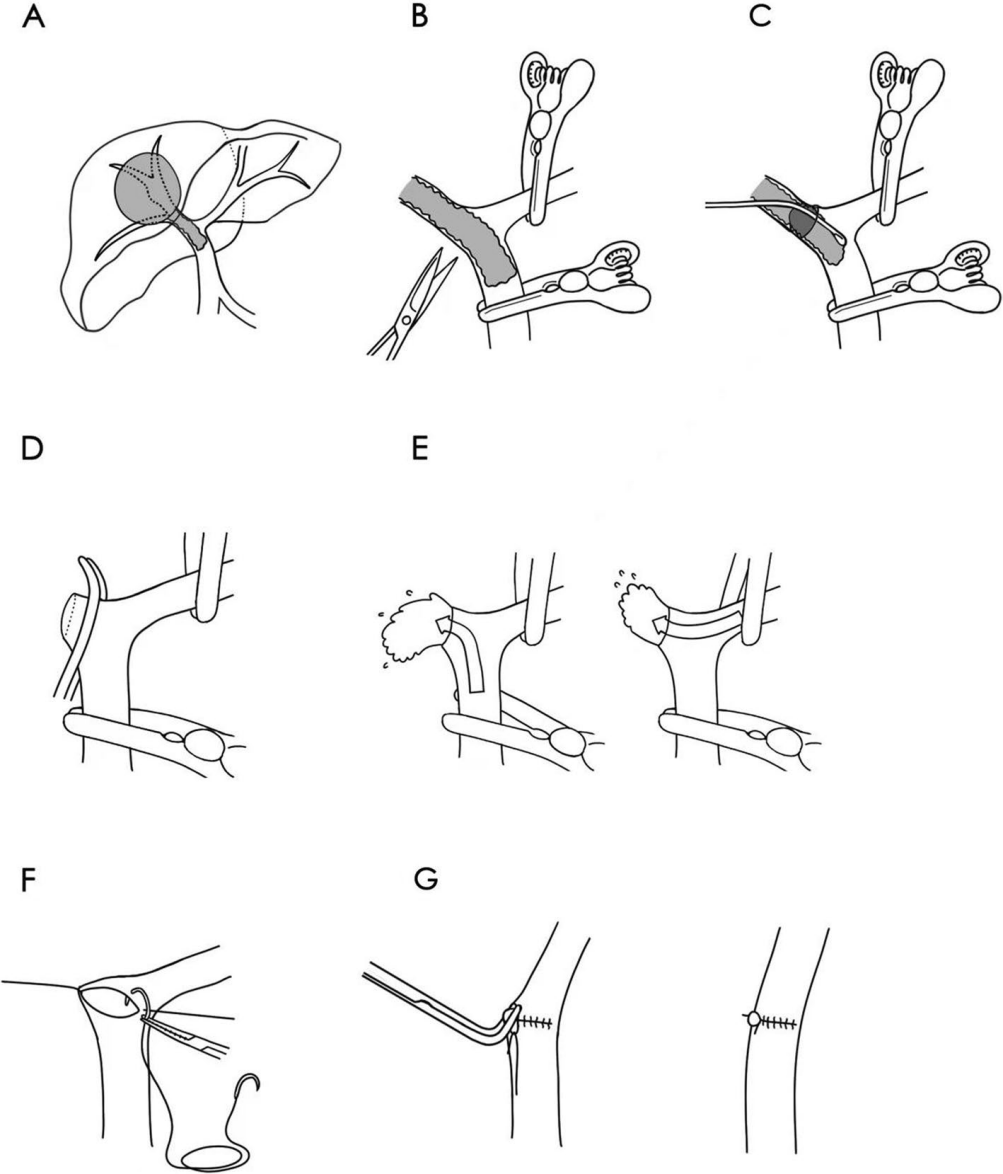
Surgical approach for VP3/4 PVTT. **A** HCC with Vp4 PVTT in right hemi liver; **B** reveal the MPV, the RPV and the LPV and temporarily block the MPV and the LPV, then longitudinally dissect the RPV; **C** completely extract the PVTT; **D** cut the RPV and occlude the cut edge; **E** flush the potential residual PVTT from the RPV and the LPV; **F** transversely suture the stump; **G** convert the angle of portal vein to straight Abbreviations: *HCC* Hepatocellular carcinoma, *Vp* portal vein invasion, *PVTT* portal vein tumor thrombus, *MPV* main portal vein, *RPV* right portal vein, *LPV* left portal vein

The modified surgical approach was explained with the right hemihepatectomy with right Vp4 PVTT as an example (Fig. 1). Technical details are as follows: Adequate exposure of the MPV, RPV, and LPV is crucial (Fig. 1A). Before longitudinally incising the portal vein, it is essential to block the main portal vein and contralateral portal vein branches thoroughly (Fig. 1B). A bile duct scraper was utilized to completely extract the PVTT (Fig. 1C). Subsequently, the RPV was incised, and the incised edge was occluded (Fig. 1D). It was imperative to flush the potentially residual PVTT by gradually releasing the blockades (Fig. 1E). Subsequently, transverse suturing was performed to enlarge the inner diameter of the portal vein, preventing portal vein stenosis (Fig. 1F). Finally, the angle of the portal vein was converted to a straight configuration, maintaining hemodynamic stability and shortening the redundant portal vein (Fig. 1G).

### Outcomes

**Fig. 2:**
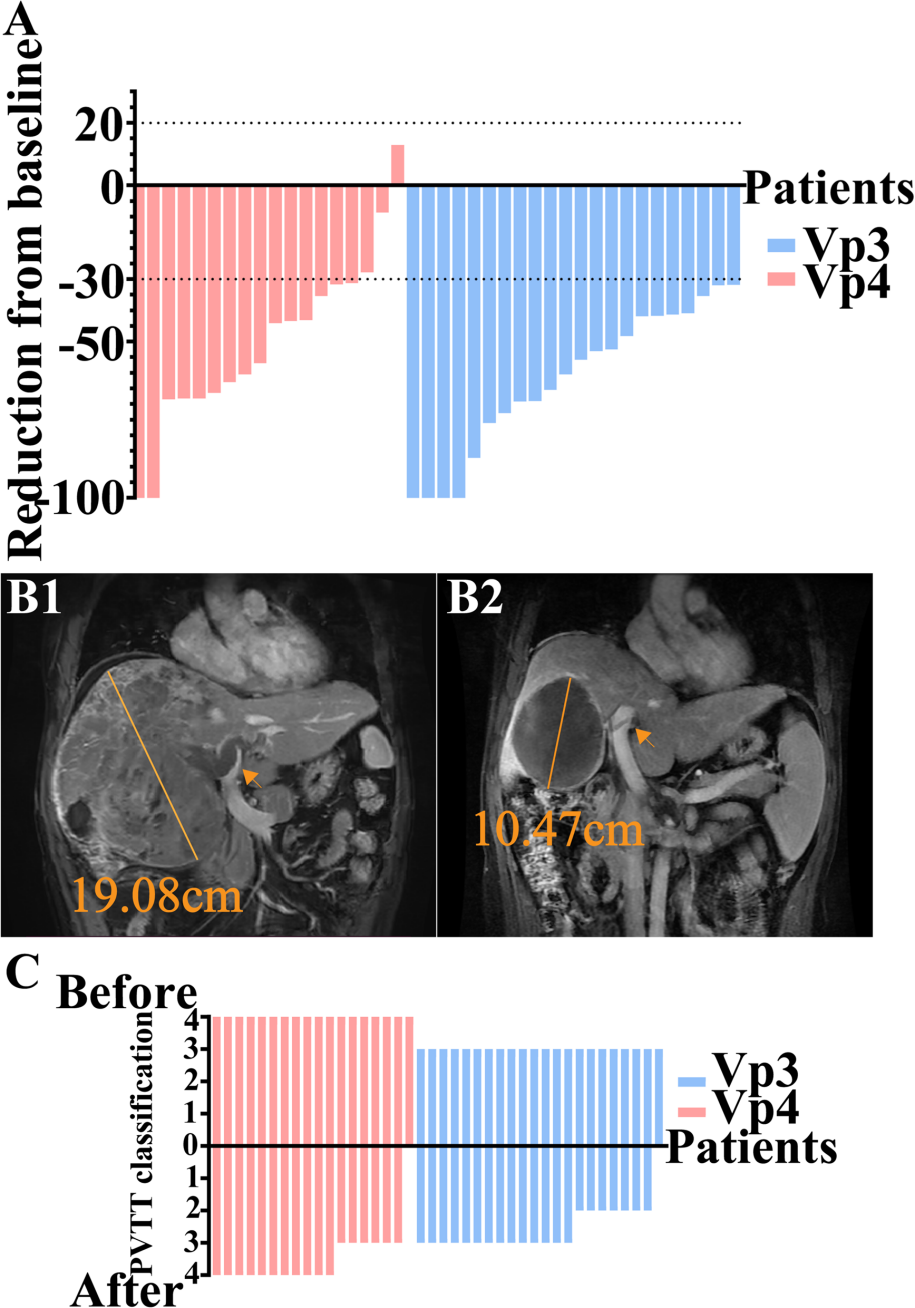
Summary of pathological and radiological evaluation. **A** The waterfall plot of main tumor reduction after combination therapy. **B**1 A 51-year-old man with a 19.08 cm diameter tumor that contained multiple enhanced lesions and Vp4 PVTT before combination therapy; **B**2 after combination therapy, the tumor was reduced to 10.47 cm with a complete response of enhanced lesions and PVTT downstaged into Vp3. **C** The waterfall plot of the PVTT classification before and after combination therapy based on radiological evaluation. Abbreviations: *PVTT* portal vein tumor thrombosis, *Vp* portal vein invasion

Table 2 summarizes the radiological, pathological and recurrence outcomes of PVTT patients. Radiological assessment of main tumor showed similar outcomes between Vp3 and Vp4 groups (*p* = 0.165): 4 CR (18.2%), 18 PR (81.8%), and 0 SD (0.0%) in Vp3 group; 2 CR (11.1%), 13 PR (72.2%), and 3 SD (16.7%) in Vp4 group. Figure 2A illustrates a waterfall plot demonstrating the reduction of the main tumor, as evaluated by the mRECIST criteria, following combination therapy in both the Vp3 and Vp4 groups. It is worth mentioning that one patient in Vp4 group exhibited a downstaging from Vp4 to Vp3 classification of PVTT, despite the absence of significant tumor regression. As for PVTT, 8 patients (36.4%) in the Vp3 group and 7 patients (38.9%) in the Vp4 group achieved downstaging, with no significant difference observed (*p* = 0.870). Figure 2C illustrates the alterations in PVTT classification before and after combination treatment in the Vp3 and Vp4 groups. Figure 2B1 shows a 51-year-old man with a 19.08 cm diameter tumor and Vp4 PVTT, and in Fig. 2B2, the tumor was reduced to 10.47 cm and PVTT down-staged into Vp3 after combination therapy.

**Table 2:** Outcomes of PVTT patients

Variables	Vp3 (*N* = 22) n (%)	Vp4 (*N* = 18) n (%)	*p* value
**Radiological outcomes**			
Radiological assessment of main tumor, per mRECIST criteria			0.165
CR	4(18.2)	2(11.1)	
PR	18(81.8)	13(72.2)	
SD	0(0.0)	3(16.7)	
Radiological PVTT downstage, yes	8(36.4)	7(38.9)	0.870
**Pathological outcomes**			
Overall pathological pCR response, yes	6(27.3)	4(22.2)	0.040
PVTT pathological pCR response, yes	14(63.6)	11(61.1)	0.870
R0 resection, yes	22(100.0)	17(94.4)	0.450
**Recurrence outcomes**			
Recurrence patterns			0.131
Intrahepatic recurrence	7(31.8)	10(55.6)	
Extrahepatic metastasis	5(22.7)	1(5.6)	
Synchronous intrahepatic and extrahepatic recurrences	1(4.5)	0(0.0)	
Recurrence treatment			0.444
Curative resection	2(9.1)	4(22.2)	
Radiofrequency ablation	3(13.6)	2(11.1)	
After-line drug therapy	3(13.6)	0(0.0)	
Best support treatment	1(4.5)	1(5.6)	
TACE + After-line drug therapy	4(18.2)	4(22.2)	

*Vp* portal vein invasion, *mRECIST* modified response evaluation criteria in solid tumors, CR complete response, *R0* no residual tumor, *TACE* transcatheter arterial chemoembolization

Pathological assessment revealed that the Vp3 group had a higher rate of overall pCR rate compared to the Vp4 group (27.3% vs 22.2%, *p* = 0.040). However, the rates of PVTT PCR were similar between the Vp3 and Vp4 groups (63.6% vs 61.1%, *p* = 0.870). R0 resection was achieved in most cases, with only one R1 resection (5.6%) in Vp4 group (*p* = 0.450).

There was no significant difference in recurrence patterns (intrahepatic vs extrahepatic vs Synchronous) between the Vp3 and Vp4 groups (*p* = 0.131). Furthermore, the treatment approaches for recurrence showed similarity between the Vp3 and Vp4 groups (*p* = 0.444).

### Follow-up

**Fig. 3:**
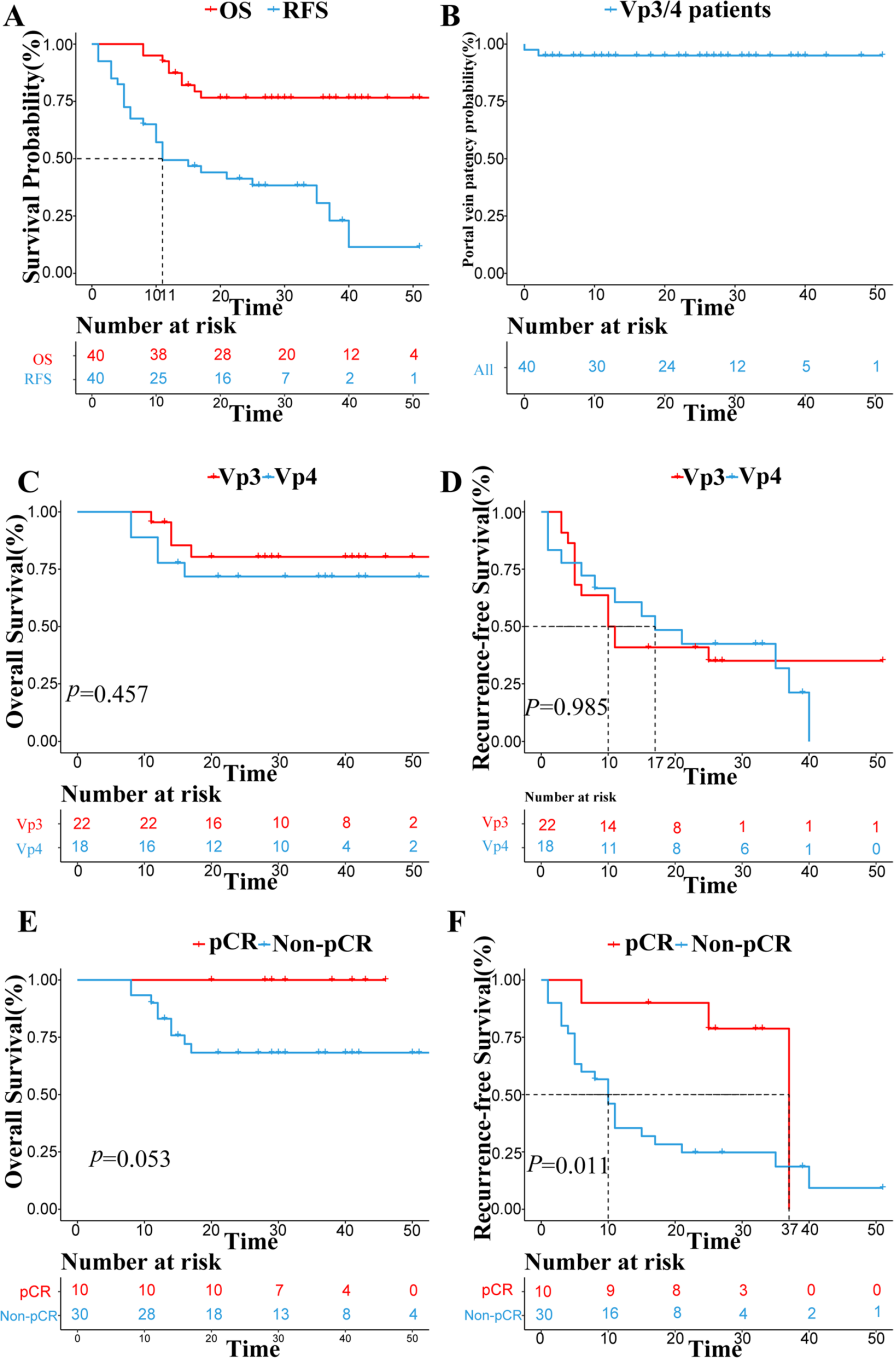
Survival analysis of Vp3/4 PVTT patients who received salvage surgery following combination therapy of TKIs and PD-1 inhibitors. **A** Overall survival and Recurrence-free survival of all cohort: the mOS had not reached, and the mRFS was 11 months (95% CI, 2.68–19.32). **B** the 3-year portal vein patency rates of all patients were 95.0%. **C** Overall survival stratified by the Vp classification (*P* = 0.457). **D** Recurrence-free survival stratified by the Vp classification (*P* = 0.985). **E** Overall survival stratified by overall pCR (*P* = 0.053). **F** Recurrence-free survival stratified by overall pCR (*P* = 0.011). Abbreviations: *PVTT* portal vein tumor thrombosis, *Vp* portal vein invasion, *mOS* median overall survival, *mPFS* median progression-free survival; *pCR* pathological complete response

The cut-off date for the present analysis was November 2023, and the median follow-up was 29.5 months (8–55 months). At the time of data collection, tumor recurrence was detected in 24 patients, and 9 patients died. As illustrated in Fig. 3A, the mOS had not reached, and the 3-year OS rates after initial treatment were all 76.6%; an mRFS of 11 months could be achieved (95% CI, 2.68–19.32), and the 1-and 3-year RFS rates after salvage surgery were 72.5% and 30.7%. As illustrated in Fig. 3B, the median portal vein patency rate of all patients had not reached, and 3-year portal vein patency rates were 95.0%.

As illustrated in Fig. 3C and D, the OS and RFS stratified by the Vp classification was not significantly different (OS *P* = 0.457, RFS *P* = 0.985), and the mRFS of Vp3 and Vp4 patients were 10 months (95% CI, 5.403–14.597) and 17 months (95% CI, 4.00–30.00), respectively. Patients with overall pCR had a longer OS (*P* = 0.053) and RFS (*P* = 0.011, HR = 0.242, 95% CI, 0.072–0.816) (Fig. 3E and Fig. 3F), and the mRFS for patients with and without overall pCR was 37 months (95% CI, NA) and 10 months (95% CI, 7.46–12.55), respectively. It is noteworthy that no patients with overall pCR experienced death during follow-up.

### Safety

The overall incidence of AEs was similar between the Vp3 and Vp4 groups, with 19 cases (86.4%) in the Vp3 group and 18 cases (100%) in the Vp4 group (*p* = 0.253). Notably, no grade 4 or higher AEs occurred in either group. The differences in the incidence of grade 3 AEs between the Vp3 and Vp4 groups were not statistically significant (*p* = 0.486). Among the grade 3 AEs, hypertension (5 patients in Vp3 group, 2 patients in Vp4 group) was the most common, followed by GGT increased, liver autoimmune disorder, blood bilirubin increased, platelet count decreased, abdominal pain, myocarditis and fever (Table 3).

**Table 3:** Combination therapy related AEs and perioperative complications

Adverse events	Vp3 (*N* = 22) n (%)	Vp4 (*N* = 18) n (%)	*p* value
**Combination therapy related AEs**			
All AEs, yes	19 (86.4)	18 (100)	0.253
Grade 3 AEs			0.486
Hypertension	5 (22.7)	2 (11.1)	
GGT increased	1 (4.5)	2 (11.1)	
Liver autoimmune disorder	0 (0.0)	1 (5.6)	
Blood bilirubin increased	0 (0.0)	1 (5.6)	
Platelet count decreased	0 (0.0)	1 (5.6)	
Abdominal pain	0 (0.0)	1 (5.6)	
Myocarditis	1 (4.5)	0 (0.0)	
Fever	0 (0.0)	1 (5.6)	
**Perioperative complications**			
Clavien-Dindo score			0.613
No or I or II	18 (81.8)	17 (94.4)	
IIIa	3 (13.6)	1 (5.6)	
IV	1 (4.5)	0 (0.0)	
PVT classification			0.439
No PVT	21 (95.5%)	15 (83.3%)	
Grade 1	0 (0.0)	2 (11.1%)	
Grade 2	1 (4.5%)	1 (5.6%)	
Long-term portal vein patency, yes	21(95.5)	17(94.4)	0.704

*Vp* portal vein invasion, *AEs* adverse events, *GGT* Gamma-glutamyl transpeptidase

There was no statistically significant difference in the distribution of Clavien-Dindo scores between the Vp3 and Vp4 groups (*p* = 0.613). One patient in the Vp3 group experienced a grade 4 complication, specifically pulmonary embolism, and required intensive care unit (ICU) transfer and anticoagulation therapy until full recovery. There was no significant difference in PVT occurrence between the Vp3 and Vp4 groups (*p* = 0.439). In the Vp3 group, 1 patient (4.5%) had a grade 2 PVT, while in the Vp4 group, 2 patients (11.1%) had grade 1 PVT and 1 patient (5.6%) had grade 2 PVT according to the Yerdel classification [28]. Following anticoagulation therapy, all grade 1 thromboses resolved in the long term. Among the 2 patients with grade 2 PVT, they showed a gradual progression towards portal cavernoma formation. However, they also developed robust collateral circulation and maintained well liver function without any symptoms. Notably, the long-term portal vein patency rates were 95.5% in the Vp3 group and 94.4% in the Vp4 group, with no significant difference observed (*p* = 0.704). No life-threatening AEs or perioperative complications were observed in either group, and they were effectively managed.

## Discussion

To date, few studies have concentrated on patients with HCC and PVTT receiving salvage surgery following combination treatment of TKIs and PD-1 inhibitors. In the present study, we reported the outcomes of 40 patients with initial unresectable HCC with VP3/4 PVTT who underwent salvage surgery following successful combination therapy, as well as the surgical experience of Vp3/4 PVTT. These findings were consistent with previous studies. Our team previously reported 35 HCC patients with major vascular invasion accepted the combination therapy, of whom 14 patients were converted successfully and underwent salvage surgery without postoperative mortality [16], and we also reported a pilot study of 10 patients who underwent salvage surgery after the combination therapy [18]. Moreover, Zhu et al. [19] conducted a cohort study on 101 patients with advanced HCC, and 24 patients underwent salvage surgery after combination treatment. Also, Yang et al*. *[20] and zhu et al. [21] both reported treating a small group of patients with this treatment approach. These studies emphasize the importance of further research to fully comprehend the characteristics of salvage surgery following combination therapy.

It is controversial whether patients with PVTT could benefit from surgery. To date, a growing body of evidence demonstrated that surgery is beneficial for selected patients with PVTT. A multicentric study from Italy compared surgery vs sorafenib for BCLC C patients and found that liver resection was followed by better OS (*p* < 0.001) and PFS (*p* = 0.007) [29]. In a systematic review conducted by Glantzounis et al*.* [30], 3 659 patients with PVTT from 29 studies were assessed, and the mOS and 3-year survival rate were 15 months and 33%. Kokudo et al*. *[31] analyzed 6 474 patients with PVTT, and it was found that the mOS in surgery group was 1.77 years longer than that in non-surgery group (*p* < 0.001), while this finding was not observed in patients with Vp4 PVTT. The mOS and mRFS of patients with Vp4 PVTT in the surgery group were 0.91 years (95% CI, 0.75–1.23) and 0.38 years (95% CI, 0.29–0.45). Moreover, Wang et al*. *[32] performed a multi-center study in China, and a total of 1 580 patients with PVTT were analyzed. According to the Cheng et al.’s classification, the mOS of patients who underwent surgery were 15.9 months (type I), 12.5 months (type II), and 6 months (type III), respectively, and surgery was found as the best treatment for patients whose PVTT did not reach the MPV. In summary, these studies showed that direct surgery can only offer limited survival benefits to patients with Vp4 whose outcome is the worst. The present study found that the mOS of patients with Vp4 PVTT after combination therapy was significantly longer compared to previous studies and even comparable to Vp3 patients.

The selection of the appropriate surgical strategy should be based on the location of PVTT [2, 33, 34]. For PVTT cases that are limited to the resection line (Vp1-2), segmental resection or hemi-hepatectomy is considered to be a viable treatment option. If PVTT that extends to or beyond the bifurcation (Vp3-4), en bloc resection with portal vein reconstruction or thrombectomy is recommended. However, the optimal method for Vp3-4 PVTT remains a topic of debate [35, 36]. Chok et al*. *[37] compared the outcomes of en bloc resection plus portal vein reconstruction and thrombectomy in patients with Vp4 PVTT and found that the mOS (9.4 months vs. 8.58 months), mRFS (3.78 months vs. 1.51 months), and recurrence patterns were comparable. Therefore, to minimize the surgical risk, thrombectomy was chosen in this study. In the suturing of the portal vein after thrombectomy, the conventional thrombectomy choosing either the closure of the stump or end-to-end anastomosis to seal the portal vein incision​​ [35, 36, 38]. This can leave an angled closure at the suture site, causing alterations in the hemodynamics within the portal vein. Taking into consideration the above factors, a novel surgical approach was employed for Vp3/4 PVTT (Fig. 1). We summarized the key aspects of the approach as "longitudinal incision and transverse suturing" and "angle-to-straight conversion". Compared with conventional PVTT thrombectomy, [35, 36, 38] this novel approach offers the following advantages: (1) PVTT following combination therapy has undergone thorough organization, reducing the likelihood of adhesion to the portal vein wall, making resection less challenging. (2) After downstaging, the resection scope is significantly reduced, with some Vp4 cases downstaged to Vp3, eliminating the need to address the MPV. (3) "Longitudinal incision and transverse suturing" effectively mitigate portal vein c problems, while "angle-to-straight conversion" further reduces the risk of PVT. In the present study, the 3-year portal vein patency rates were up to 95.0%, the modified surgical approach can complete removal of PVTT and ensures long-term patency of the portal vein. Additionally, this approach presents some potential challenges: a considerable amount of experience in vascular surgery is required for the anatomy of the hepatic hilum, the portal vein suturing technique and the conversion of the portal vein angle.

There are several special challenges of the salvage surgery following combination therapy that are summarized as follows: (1) The administration of TKIs, which are VEGFR blockers, may increase the risk of perioperative bleeding and prolong the surgical incision healing period [21]. To mitigate these risks, TKIs were stopped 7 days prior to surgery. (2) an immune-related inflammation in the liver could be induced by PD-1 inhibitors, making it more fragile [21]; (3) the size of tumor was relatively huge and remnant liver volume was close to the extreme. Thus, the definition of tumor resection margin may not be suitable for radical resection and an open surgery is approach preferable; 4) The occurrence of portal vein thrombosis is relatively high, so it's important to regularly detect any complications as early as possible. In our study, there was no mortality during perioperative period, indicating that this treatment strategy is relatively safe.

Patients with pCR are strongly accompanied with a longer OS and RFS, which is consistent with previously reported results [19, 39, 40]. Zhu et al*. *[19] reported that 24 patents received salvage surgery after combination treatment, and among them, 10 patients achieved pCR, who had a favorable RFS compared to patients with non-pCR. Furthermore, Allard et al*. *[40] found that survival was longer in patients who had less than 10% of viable cancer cells remaining, which may refer to the Major pathological response (MPR) of HCC. However, the findings regarding MPR as a predictor of survival in HCC are still unknown, and additional research is needed.

However, there are several limitations in the present study. First, the retrospective study design and a relatively small sample size might limit the levels of evidence, but a trend has been identified in the advantages of this novel approach. Second, the follow-up period was not long enough to calculate mOS and 5-year survival rate. Third, this study may have some potential biases (such as excluding non-responsive patients, or those who voluntarily abandoned surgery, being a single-center study, and comprising entirely Han Chinese individuals) and confounding factors (such as the lack of complete uniformity in the combined treatment, variations in the extent of liver, and the absence of blinding). Despite implementing measures such as ensuring all surgeries were performed by the same surgeon and making efforts to collect comprehensive imaging and follow-up data to mitigate the impact of these limitations, they may still affect the generalizability of the results. Hence, multi-center, double-blind, randomized studies with a larger sample size and different races are required to further clarify the therapeutic efficacy of combination therapy and salvage surgery.

## Conclusions

In summary, these outcomes suggested that the combination therapy of TKIs plus PD-1 inhibitors is an effective and safe treatment strategy for patients with PVTT; the modified surgical approach enables complete removal of PVTT and ensures long-term patency of the portal vein; following this treatment strategy, patients of Vp4 group can also achieve comparable outcomes to those of Vp3 patients. Finally, it is worth noting that further research should aim to identify accurate non-invasive biomarkers for the dynamic assessment of tumor and PVTT necrosis. This will guide us in selecting the most appropriate timing for salvage surgery.

## Data Availability

The datasets analyzed during the current study are not publicly available due to protecting patient privacy, but are available from the corresponding author on reasonable request.

## Abbreviations

HCC: Hepatocellular carcinoma
PVTT: Portal vein tumor thrombus
BCLC classification: The Barcelona Clinic Liver Cancer classification
mOS: Median overall survival
PD-1 inhibitors: Programmed cell death protein 1 inhibitors
TKIs: Tyrosine kinase inhibitors
mPFS: Median progression-free survival
ORR: Objective response rate
AASLD: The American Association for the Study of Liver Diseases
ECOG PS score: Eastern Cooperative Oncology Group performance status score
mRECIST: The modified Response Evaluation Criteria in Solid Tumors
DCE-MRI: Dynamic contrast-enhanced magnetic resonance imaging
CR: Completed response
PR: Partial response
SD: Stable disease
pCR: Pathological complete response
AFP: Alpha-fetoprotein
MPV: Main portal vein
RPV: Right portal vein
LPV: Left portal vein
